# Aphorisms Respecting the Rodent Ulcers

**Published:** 1861-01

**Authors:** 


### *Aphorisms Respecting the Rodent Ulcers.*

1. That there occurs not infrequently on one or other part of the face a form of ulceration which is characterized by an indurated edge, and by a tendency to spread to adjacent structures, without regard to difference of tissue; which is very slow in its progress; does not cause much pain; does not induce cachexia, and is never followed by enlarged glands or deposits in the viscera.*

2. Sections of the indurated edge of this ulcer (or of the portions of new growth which are sometimes produced about it) do not exhibit the cell-structures met with in epithelial or scirrhous cancer, but only those of organizing fibrous tissue.

3. This ulcer differs from lupus exedens in that it never occurs in the young, and never gets well spontaneoulsy, while lupus exedens but rarely begins after the age of thirty,

---

* In making this assertion, I am borne out by all the facts hitherto recorded. Fully acknowledging, however, the near relationship of rodent ulcer to cancer, I have but little doubt that it will now and then so far deviate from its usual course as to affect the glands, and quite anticipate in the future to hear of such a case. Epithelial cancer may be said to almost never affect the internal organs, yet a few cases are on record in which it has done so. Such exceptions, however, only prove the general rule; and just as the epithelial cancer very exceptionally affects the viscera, so will rodent very exceptionally affect the lymphatics. Professor Langenbeck has mentioned to me a case in which he excised a rodent ulcer from the side of a woman's nose, who aftewards remained well for nine years, and was then attacked by cancer of the uterus, followed by secondary growth and death. Such a fact is, however, very different from one in which the cancerous infection should advance, as in other malignant disease, through the lymphaeic system, from the original ulcer.

and usually tends after the lapse of time to cicatrize spontaneously. The two, also, further differ, in that lupus has a tuberculated, inflamed border, whithout any great degree of induration; while the edge of the ulcer in question presents an extremely indurated ridge, without tubercles, and comparatively free from inflammatory congestion.

4. The ulcer in question differs from cancer in that there is but seldom present any tendency to the production of new material, that it never causes the glands to enlarge nor induces morbid growths in the internal viscera.

5. Although it must be freely admitted that this disease is closely allied to cancer, and that in its inveteracy under treatment, and its tendency if not removed to spread deeply and extensively, it well deserves the designation of "locally malignant," yet it is inconvenient in practice to call it "cancer of the skin," since there are other forms of cutaneous cancer (the epithelial, scirrhs, melanotic, etc.) essentially different from it, and of a far higher degree of malignancy.

6. The term "a peculiar ulcer occuring in the eyelids," is too vague, and also involvs an erroneous statement as to uniformity of location, an objection which, also, in addition to what has been stated above, applies to "cancer of the eyelids," since this ulcer is met with on many other parts besides the palpebræ.

7. To the designation of rodent ulcer given to this disease by Lebert, and adopted in this county by Paget (see Lectures on Surgical Pathology), no objection applies, excepting that it is more vague than desirable. Of those in use it is certainly the best, and should the disease become generally recognized by the Profession under that name, the vagueness of its meaning will by custom soon cease.

8. The rodent ulcer is most commonly met with between the ages of 50 and 60, and is equally frequent in the two sexes.

9. It occurs but very rarely on any other region than the integument of the face, and is most common in the eyelids.

10. It is a singular and very significant fact that no case has yet been recorded in which the rodent ulcer attacked the lower lip, either primarily or by extension, while that part is well known to be a very frequent seat of epithelial cancer.

11. The *diagnosis* of rodent ulcer is usually easy. An ulcer with a hard sinuous edge, situated on some part of the skin of the upper two-thirds of the face, of several, or, perhaps, many years' duration, almost painless, and occurring in a middle-aged or elderly person, of fair health and without enlarged glands—such a sore is almost certain to be of the rodent type.

12. The *prognosis* of rodent ulcer varies with the stage of the disease and the treatment it is intended to pursue. If left to itself, it will slowly but surely advance both in extent and depth, and will probably destroy the patient's life in the course of from ten to twenty-five years, death being eventually produced by the exhaustion consequent on suppuration, hemorrhages, pain, etc., and very probably aggravated by inability to take sufficient food owing to the diseased state of the mouth. If the case can be seen in an early stage, while complete removal either by knife or escharotics is practicable, a favorable opinion may be given as to the probable non-return of the disease. The younger the patient, the more rapid will be the course of the disease, and *vice versa;* and the younger the patient the more nearly is the disease allied to cancer, and the more likaly to recur after removal.

13. The only treatment which the rodent ulcer admits of is local, and the best is that which obtains its freest removal with the least injury to the parts concerned. In some localities, and in some stages, escharotics, such as the chloride of zinc, may be advisable, but in most excision and transplantation of skin is the more certain and satisfactory.

14. A widely-diffused knowledge of the true pathology of rodent ulcer may be expected to result in considerable advantage to the sufferers from that disease, since it will encourage to the early and free adoption of local measures and to the employment of excision and transplantation, even in some cases which, if considered cancerous, would certainly be beyond relief by surgical art.—*Med. Times & Gazette.*

